# The Prospective Associations of Lipid Metabolism-Related Dietary Patterns with the Risk of Diabetes in Chinese Adults

**DOI:** 10.3390/nu14050980

**Published:** 2022-02-25

**Authors:** Qi Liu, Qiaorui Wen, Jun Lv, Zumin Shi, Yu Guo, Pei Pei, Huaidong Du, Ling Yang, Yiping Chen, Xiaofang Zhang, Dan Schmidt, Sam Sansome, Junshi Chen, Canqing Yu, Zhengming Chen, Liming Li, on behalf of the China Kadoorie Biobank (CKB) Collaborative Group

**Affiliations:** 1 Department of Epidemiology and Biostatistics, School of Public Health, Peking University, Beijing 100191, China; liuqi.mun@foxmail.com (Q.L.); wqr119@163.com (Q.W.); epi.lvjun@vip.163.com (J.L.); lmleeph@vip.163.com (L.L.); 2 Key Laboratory of Molecular Cardiovascular Sciences, Ministry of Education, Peking University, Beijing 100191, China; 3 Peking University Center for Public Health and Epidemic Preparedness & Responses, Beijing 100191, China; 4 Human Nutrition Department, College of Health Sciences, QU Health, Qatar University, Doha 2713, Qatar; zumin@qu.edu.qa; 5 National Center for Cardiovascular Diseases, Fuwai Hospital Chinese Academy of Medical Sciences, Beijing 100730, China; guoyu@kscdc.net; 6 Chinese Academy of Medical Sciences, Beijing 100730, China; peipei@kscdc.net; 7 Medical Research Council Population Health Research Unit, University of Oxford, Oxford OX3 7LF, UK; huaidong.du@ndph.ox.ac.uk (H.D.); ling.yang@ndph.ox.ac.uk (L.Y.); yiping.chen@ndph.ox.ac.uk (Y.C.); 8 Clinical Trial Service Unit and Epidemiological Studies Unit (CTSU), Nuffield Department of Population Health, University of Oxford, Oxford OX3 7LF, UK; dan.schmidt@ndph.ox.ac.uk (D.S.); sam.sansome@ndph.ox.ac.uk (S.S.); zhengming.chen@ndph.ox.ac.uk (Z.C.); 9 Maiji Center for Disease Control and Prevention, Tianshui 741020, China; zhangxiaofangmjcdc@foxmail.com; 10 NHC Key Laboratory of Food Safety Risk Assessment, China National Center for Food Safety Risk Assessment, Beijing 100022, China; chenjunshi@cfsa.net.cn

**Keywords:** dietary patterns, reduced rank regression, lipid metabolism, diabetes

## Abstract

Background: This study aimed to identify lipid metabolism-related dietary patterns with reduced rank regression (RRR) among Chinese adults and examine their associations with incident diabetes. Methods: We derived lipid metabolism-related dietary patterns using an RRR with 21 food groups as predictors as well as total cholesterol, low-density lipoprotein (LDL) cholesterol, high-density lipoprotein (HDL) cholesterol, triglycerides, body mass index (BMI), and waist circumference from the responses of 17,318 participants from the second resurvey of the China Kadoorie Biobank (CKB). The dietary scores were calculated for the entire cohort. We followed up 479,207 participants for diabetes incidence from the baseline and used multivariable Cox regression models to estimate the hazard ratios (HRs) and 95% confidence intervals (CIs). Results: Two lipid metabolism-related dietary patterns were extracted. The dietary pattern—characterized by high intakes of fish, poultry, and other staples as well as fresh fruit and vegetables—was correlated with a higher BMI, waist circumference, and LDL cholesterol. Participants in the highest quintile (Q5) had a 44% increased risk of diabetes incidence when compared with those in the lowest quintile (Q1) (HR = 1.44; 95% CI: 1.31–1.59). Conclusions: A dietary pattern characterized by high intakes of both animal and plant foods was related to obesity and dyslipidemia and could increase the risk of diabetes incidence.

## 1. Introduction

Diabetes is a chronic disease that occurs either when the pancreas does not produce enough of the blood sugar-regulating hormone insulin or when the body cannot effectively use the insulin it produces. Hyperglycemia, or elevated blood sugar, is a common effect of diabetes that eventually leads to serious damage to many of the systems of the body, especially the nerves and blood vessels [1]. Findings from previous studies have shown that lifestyle interventions such as diet and exercise could lead to various metabolic benefits, including reduced weight, improved lipid profiles, decreased diabetes incidence, and improved glycemic control in those with diabetes [2]. According to dietary analyses over the past decade, high intakes of processed meat [3], white rice [4], and fish [5] could increase the risk of diabetes. An exploration analysis identified several well-known healthy dietary patterns, e.g., the Mediterranean diet and Dietary Approaches to Stop Hypertension (DASH). Compared with single foods or nutrients, dietary patterns are more practical in dietary interventions because they are much closer to the daily dietary habits of an individual [6]. However, neither a priori nor exploratory approaches (e.g., principal component, factor, and cluster analyses) could utilize the information of the biological pathways for diseases and the underlying dietary data. Hence, hybrid approaches such as the reduced rank regression (RRR) model have been introduced to identify dietary patterns that are more related to the disease outcomes of interest [7,8].

RRR is a statistical method that determines the linear functions of the predictors (food groups) by maximizing the explained variation in the response variables (disease-related biomarkers) [9]. Existing evidence shows that plasma lipids [10] and adiposity indicators (e.g., an elevated BMI and waist circumference) [11] are risk factors for diabetes. However, to our knowledge, evidence of lipid metabolism-related dietary patterns as well as their associations with diabetes is limited, especially among Chinese adults. Therefore, the present study aimed to identify lipid metabolism-related dietary patterns using plasma lipids and indicators of being overweight or obesity as the response variables with the RRR method. We then examined the association between dietary patterns and diabetes among Chinese adults.

## 2. Materials and Methods

### 2.1. Study Population

The CKB was a general population-based prospective cohort study that enrolled approximately 0.5 million adults aged 30–79 years in China. Details of the study design and profile have been previously reported [12,13]. In brief, a total of 512,725 individuals (59% women) aged 30–79 years were recruited during 2004–2008 from 10 geographically diverse areas (5 urban areas and 5 rural areas) across China. Since the initial recruitment, a random sample (~5%) of the surviving participants in the CKB has been periodically resurveyed using the same procedures as the baseline. The first and the second resurveys were undertaken in 2008 and 2013, respectively.

For the derivation of dietary patterns using a reduced rank regression (RRR), we involved participants from the second resurvey of the CKB but excluded participants with self-reported diabetes (*n* = 2409), cancer (*n* = 293), or statins taken (*n* = 140) to reduce their effects on the diet–lipid relationship. We also excluded participants without responses (total cholesterol, HDL cholesterol, LDL cholesterol, triglycerides, BMI, and waist circumference) (*n* = 5056) or without dietary data (*n* = 23), resulting in a final number of 17,318 participants for the analyses.

For the analyses of the derived dietary patterns and diabetes, we involved all participants in the entire cohort but excluded those with a history of diabetes (*n* = 30,006) or cancer (2578) at the baseline survey. In addition, to avoid reverse causality, participants with a current medication of statins (*n* = 932) were also excluded because those individuals could have changed their diet. This resulted in 479,207 participants (197,264 men and 281,943 women) available for the present analyses.

The CKB study was approved by the ethical committee and research council of the Chinese Centre for Disease Control and Prevention and the Oxford Tropical Research Ethics Committee at the University of Oxford. Prior to the survey, all participants provided written informed consent.

### 2.2. Dietary Assessment

Habitual dietary intake information over the previous 12 months was assessed using a frequency questionnaire containing 12 major food items in China: rice, wheat, other staples (such as corn and millet), meat, poultry, fish, eggs, fresh vegetables, fresh fruit, preserved vegetables, soybeans, and dairy products. The five frequency levels of habitual consumption (i.e., never/rarely, monthly, 1–3 days/week, 4–6 days/week, or daily) were recorded into days/week: 0, 0.5, 2, 5, and 7, respectively. In the second resurvey, the dietary questionnaire also asked about the daily intake of each food group, which enabled us to estimate the average consumption at the baseline for each of the five frequency categories. The weekly amount of food consumption and daily energy intake were then calculated for each participant at the baseline and the second resurvey.

In addition, data regarding the frequency and number of beverages consumed were collected at the baseline and resurveys, including five types of alcohol (beer, rice wine, wine, heavy spirits (≥40%), and light spirits (<40%)) and four types of tea (green/jasmine tea, oolong tea, black tea, and other tea). Thus, the average amount of each beverage consumed per week was calculated [14].

The CKB questionnaire was repeated among a subsample of 926 participants within one year after the baseline and showed a good reproducibility of the food and beverage items.

### 2.3. Measurements of the BMI, Waist Circumference, and Plasma Lipids

Trained health staff conducted the physical examination for the CKB study. Standing height was measured to the nearest 0.1 cm without shoes using a portable stadiometer and weight was measured to the nearest 0.1 kg using the scale function of the TBF-300 body composition analyzer (Tanita Inc., Tokyo, Japan). The BMI was calculated as the weight in kilograms divided by the height in meters squared. The waist circumferences were measured to the nearest 0.1 cm with a non-stretchable tape measure. In the second resurvey, the plasma lipid concentrations were measured using AU680 Chemistry Analyzers (Beckman-Coulter, Brea, CA, USA), which provided direct homogenous assays for LDL cholesterol and HDL cholesterol as well as enzymatic color assays for the total cholesterol and triglycerides [15].

### 2.4. Outcome Ascertainment

All the CKB participants were followed up through death registries, disease surveillance, health insurance records, and active confirmations. Trained staff, blinded to the baseline information, coded all the cases with the International Classification of Diseases, 10th revision (ICD-10) [13]. In the present study, incident diabetes cases were identified by E10–E14. For the current analyses, we updated the morbidity data until 31 December 2017.

### 2.5. Covariate Assessment

At the baseline, trained staff using a comprehensive laptop-based questionnaire collected information on the socio-demographic status (e.g., age, gender, region, education status, household income, and marital status), lifestyle factors (e.g., smoking, alcohol drinking, and physical activity), and family medical history. Physical activity was estimated as the sum of the metabolic equivalent hours per day (MET-hours/day) based on the usual type and duration of activities related to work, commuting, household chores, and leisure time exercise during the previous 12 months [16].

### 2.6. Statistical Analyses

The present study included two steps. Firstly, we derived lipid metabolism-related dietary patterns using an RRR from 17,318 participants from the second resurvey. We then calculated the dietary scores for each participant at the baseline (*n* = 479,207) and examined the associations between the dietary patterns and diabetes.

In the RRR analysis, the predicting variables were 12 food groups and 9 types of beverage. The response variables were the log-transformed lipid indicators (i.e., total cholesterol, HDL cholesterol, LDL cholesterol, and triglycerides) and adiposity measures (BMI and waist circumference). The number of factors extracted using the RRR was determined by the proportion of explained variances in the responses (>1%) for the subsequent analyses. Food and beverage groups with absolute factor loadings > 0.30 were used to characterize the corresponding dietary pattern. Pearson’s correlation coefficients were used to assess the relationship between the dietary pattern scores and responses. The dietary pattern scores for the entire cohort were calculated as the linear combination of all 21 weighted standardized food and beverage groups with the model effect weight as coefficients, which were then used to categorize the participants into five groups according to their quintiles (Q1 to Q5, in ascending order).

The distributions of the socio-demographic status, lifestyle factors, and family history of diabetes were compared across the quintiles of the dietary pattern scores; the characteristics were expressed as means for the continuous variables and percentages for the categorical variables, which were adjusted for region, sex, and age using logistic and linear regressions, respectively. A trend test was performed by assigning median values to the quintile categories of each dietary pattern in the corresponding model.

In the prospective analysis, a Cox proportional hazard regression was used to estimate the hazard ratios (HRs) and 95% confidence intervals (95% CIs) for the risk of diabetes incidence across the quintiles of the dietary pattern scores with the lowest quintile as the reference category. The models were stratified by year of birth (5 years in each category) and sites (10 region sites) with age as the timescale. Model 1 was adjusted for gender, education attainment (no formal school, primary school, middle school, high school, or college/university), marital status (yes or no), and household income (<2500, 2500–4999, 5000–9999, 10,000–19,999, 20,000–34,999, or ≥35,000 CNY/year). Model 2 was additionally adjusted for smoking status (never, occasional, former, or current), alcohol consumption (never regular drinkers, ex-regular drinkers, occasional drinkers, monthly drinkers, reduced drinkers, or weekly drinkers), physical activity (MET-h/day), total energy intake (kcal/day), and family history of diabetes (yes or no). A test for a trend across the quintiles was performed by entering the median values of the quintile categories of each dietary pattern into the models.

To access the homogeneity of the risk estimates, we conducted stratified analyses for gender (male or female), age (<50 years or ≥50 years), urban or rural, southern or northern, current smoking status (yes or no), physical activity status (low, medium, or high tertile of MET-h/day), BMI (<24.0, 24.0–28.0, or ≥28.0 kg/m^2^), and central obesity (yes (waist–hip ratio (WHR) ≥ 0.90 for males or WHR ≥ 0.85 for females) or no (WHR < 0.90 for males or WHR < 0.85 for females)) based on Model 2. Furthermore, we accessed the interactions between dietary patterns and the stratified variables mentioned above by adding cross-product terms into Model 2. In the sensitivity analyses, we excluded participants with incident diabetes in the first two years of follow-up to exclude reverse causality.

We used Stata software version 15 for the statistical analyses. In order to derive lipid metabolism-related dietary patterns, we installed the module from within Stata by typing “ssc install rrr” [17]. Two-sided *p*-values < 0.05 were considered to be statistically significant for the primary and sensitivity analyses. For the multiplicative interaction tests in the stratified analyses, the statistical significance was defined as *p* < 0.006 (8 stratified variables were included).

## 3. Results

In the present study, two lipid metabolism-related dietary patterns were extracted with the RRR method, explaining 4.23% and 1.44% of the variation of response variables from 17,318 participants from the second resurvey of the CKB. The characteristics of these participants are shown in Table S1. The first lipid metabolism-related dietary pattern (dietary pattern-1) explained 10.56% of the variation in the total cholesterol, 8.47% in the LDL cholesterol, and 4.04% in the HDL cholesterol. In comparison, the second lipid metabolism-related dietary pattern (dietary pattern-2) explained 2.76%, 2.25%, 1.60%, and 1.42% of the variation in the BMI, waist circumference, HDL cholesterol, and LDL cholesterol, respectively. Dietary pattern-1 was characterized by high intakes of rice and low intakes of wheat. The dietary pattern score was positively correlated with the total cholesterol, LDL cholesterol, and HDL cholesterol (*p* < 0.001). Dietary pattern-2 was characterized by a high intake of fish, poultry, other staples, fresh fruit, and vegetables. This dietary pattern score was positively correlated with the BMI, waist circumference, and LDL cholesterol as well as negatively correlated with the HDL cholesterol (*p* < 0.001) (Table 1). The mean intakes of the food and beverage groups according to the quintiles of the two dietary patterns in the second resurvey and the entire cohort of the CKB are presented in Tables S2 and S3.

Compared with the participants in the lowest quintile of dietary pattern-1 (Table 2), those with higher dietary pattern scores were more likely to be female and from urban and southern areas with a higher education attainment and a higher household income; these participants were less likely to be current smokers or weekly drinkers from both the second resurvey and the entire cohort. Participants with higher scores in dietary pattern-2 were younger, lived in northern areas, and were less likely to be female.

We followed up 479,207 participants for a mean of 10.7 years (5,133,146 person–years of follow-up) and observed 18,677 cases of incident diabetes. A higher score of dietary pattern-2 was significantly associated with an increased risk of incident diabetes in both the socio-demographic adjusted model (Q5 vs. Q1 HR = 1.50; 95% CI: 1.36–1.64) and the full adjusted model (Q5 vs. Q1 HR = 1.44; 95% CI: 1.31–1.59) (Table 3). However, we did not observe any significant association between dietary pattern-1 and diabetes incidence in the full adjusted model (all *p*-value > 0.05). In the sensitivity analyses, we excluded cases occurring in the first two years of follow-up and the results did not change significantly (Table S4).

**Figure 1:**
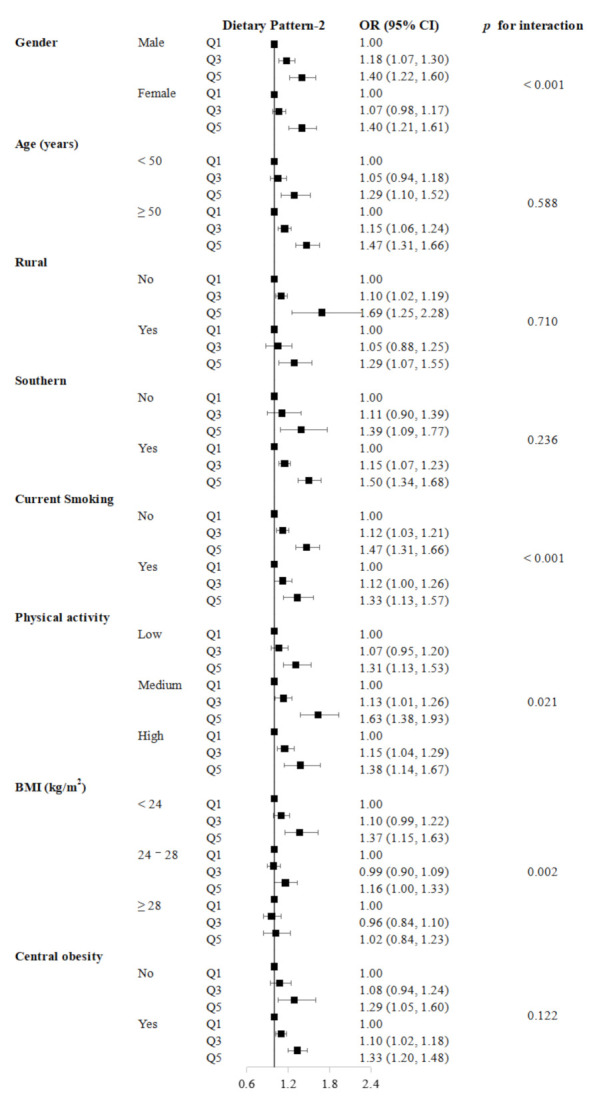
Hazard ratios (HRs) and 95% confidence intervals (CIs) of diabetes incidence according to the quintiles of dietary pattern-2 from stratified analyses from the China Kadoorie Biobank (*n* = 479,207).

In the subgroup analyses, there was a statistical interaction between dietary pattern-1 and the age of diabetes incidence (*p* for interaction < 0.001). When stratified by living region, the association between dietary pattern-1 and diabetes was stronger in participants living in urban regions (*p* for interaction < 0.001) (Table S5). The association between dietary pattern-2 and diabetes was stronger in males (*p* for interaction < 0.001). The effect of dietary pattern-2 was different when stratified by the smoking status (*p* for interaction < 0.001). As for the BMI, the association was strongest in the BMI < 24.0 kg/m^2^ group (Q5 vs. Q1 HR = 1.37; 95% CI: 1.15–1.63) than the other two groups (*p* for interaction = 0.002) (Figure 1 and Table S6).

## 4. Discussion

In the present analyses, two lipid metabolism-related dietary patterns were derived from an RRR from the CKB study. One dietary pattern—characterized by higher intakes of fish, poultry, other staples, fresh fruit, and vegetables (which predicted a higher BMI, waist circumference, and LDL cholesterol as well as a lower HDL cholesterol)—was associated with a substantially higher risk of incident diabetes. However, the other dietary pattern—characterized by high intakes of rice and positively correlated with the total cholesterol, LDL cholesterol, and HDL cholesterol—was not significantly associated with a diabetes risk.

The two dietary patterns derived from our study could explain 2.25–10.56% of the variance in the total cholesterol, LDL cholesterol, BMI, and waist circumference. The proportions were comparable with previous RRR studies using biomarkers as responses [18,19], which tended to explain less of a variation in the responses than dietary patterns with nutrients as responses because the biomarkers were more remote variables in the dietary patterns analysis [9,20].

The dietary pattern in our analysis, characterized by high intakes of both animal foods (e.g., poultry and fish) and plant foods (e.g., other staples, fresh vegetables, and fruit), was related to an increased BMI, waist circumference, and LDL cholesterol as well as a decreased HDL cholesterol. Previous studies have reported similar results. A cross-sectional study among middle-aged Chinese adults found that participants following an animal food pattern were at a higher risk of abdominal obesity [21]. Both high total and animal protein were reported to be positively correlated with the subsequent body weight gain [22]. Evidence from previous studies has also shown that high intakes of total and animal protein might increase the risk of dyslipidemia [22,23], characterized by high concentrations of triglycerides and LDL cholesterol accompanied by decreased HDL cholesterol concentrations [24]. Furthermore, the other dietary pattern derived from our study (characterized by high intakes of rice) was positively correlated with plasma cholesterols, which was consistent with previous studies. Both population studies and experiments have shown that high intakes of refined grains, mainly white rice, might increase the levels of total and LDL cholesterol as well as lower the HDL cholesterol level. However, the associations between the rice intakes and cholesterol concentrations were not significant [25,26].

In the present study, we found that participants with a higher score of the dietary pattern of high intakes of fish, poultry, other staples, fresh fruit, and vegetables had a higher risk of diabetes incidence, mainly because of the elevated BMI, waist circumference, and LDL cholesterol as well as the low plasma level of HDL cholesterol. The results were generally consistent with the findings of previous studies. As two of the most critical risk factors of diabetes, being overweight or obesity are closely related to diabetes; therefore, the common indicators of obesity (e.g., the BMI and waist circumference) could serve as intermediate biomarkers in the analyses between the dietary pattern and diabetes [27]. A previous meta-analysis by Abdullah et al. [28] combined 18 prospective cohort studies and showed that obesity (HR = 7.19; 95% CI: 5.74–9.00) and being overweight (HR = 2.99; 95% CI: 2.42–3.72) were both associated with a higher risk of diabetes compared with participants with a normal weight. Characterized by hypertriglyceridemia as well as low levels of HDL cholesterol and the presence of LDL particles, dyslipidemia is one of the most critical risk factors for diabetes, and the goal of diabetes prevention and therapy is to lower the plasma levels of non-HDL cholesterol [29]. As for the major food groups in this dietary pattern, high intakes of fish could significantly increase the risk of diabetes mellitus [5]. High intakes of total protein, especially animal protein from poultry and fish, were also confirmed to increase the risk of diabetes in a previous meta-analysis [30]. However, there were several other food groups containing plant protein in this dietary pattern (such as fresh fruit, vegetables, and other staples) that were inversely associated with diabetes in previous research [31,32]. This illustrates that the dietary pattern may also have a few health benefits on the disease outcome due to food components rich in plant protein that affect pathways other than the lipid metabolism-related one. Therefore, the characteristics of food intakes in lipid metabolism-related dietary patterns as well as their real effects on diabetes need to be studied further.

A previous meta-analysis showed a higher white rice intake could lead to a significantly elevated risk of type 2 diabetes, especially among Asian populations [4]. In our study, dietary pattern-1—characterized by high intakes of rice—was related to elevated plasma cholesterol but not significantly associated with a diabetes risk. Such an inconsistency could be explained by mechanisms other than the lipid metabolism pathways, e.g., a high dietary glycemic load [33] as well as low-benefit nutrients such as insoluble fiber, magnesium, vitamins, lignans, phytoestrogens, and phytic acid, which are lost during the refining process [34]. In addition, the consumption of eggs, alcohol, or tea was not related to the levels of blood lipids in our analysis, which indicated that the link between these food and diabetes was not due to their effect on the blood lipids in the CKB population. According to a previous study from the CKB, the components of eggs other than dietary cholesterol could have a favorable effect on health such as egg-derived phospholipids and high-quality egg protein [35], which was consistent with our study.

Our study was the first and the most extensive prospective cohort study on lipid metabolism-related dietary patterns using an RRR in China. However, several limitations require mentioning. Firstly, the dietary data only included 12 major food groups in our study; other uncollected food groups might also affect lipid levels as well as the risk of diabetes. Secondly, the dietary data collected at the baseline of the CKB only included frequency categories. However, the second resurvey questionnaires included both frequency and quantity. We estimated the food amount at the baseline accordingly but similar results were observed. Thirdly, we could not avoid the effects of changes in dietary intakes over the time of the follow-up; we also did not take the effects of different cooking methods into consideration in our study. Finally, although we excluded participants with diabetes, cancer, or current statin medication before the analyses and adjusted for a series of covariates, we still could not completely avoid residual confounding or unknown confounders.

## 5. Conclusions

In conclusion, we derived two lipid metabolism-related dietary patterns, reflecting the level of plasma lipids and indicators of being overweight or obesity in Chinese adults. High intakes of both animal foods (e.g., poultry and fish) and plant foods (e.g., other staples, fresh vegetables, and fruit) could cause obesity and dyslipidemia, thus increasing the risk of diabetes incidence. Therefore, individuals at a high risk of diabetes should follow a dietary pattern of lower intakes of nutrients such as dietary protein.

## Figures and Tables

**Table 1:** Factor loading matrix of dietary patterns derived from a reduced rank regression from 17,318 participants from the second resurvey of the China Kadoorie Biobank.

Food or Beverage Group	Dietary Pattern-1	Dietary Pattern-2
Rice	0.31	−0.23
Wheat	−0.79	0.24
Other staples	−0.16	0.36
Meat	0.28	0.15
Poultry	0.22	0.38
Fish	0.22	0.43
Eggs	−0.12	−0.01
Fresh vegetables	0.02	0.32
Fresh fruit	0.04	0.34
Preserved vegetables	−0.04	0.08
Soybeans	0.08	0.22
Dairy products	0.17	0.11
Beer	−0.07	0.15
Rice wine	0.04	0.04
Wine	0.01	0.02
Heavy spirits (≥40%)	−0.09	−0.08
Light spirits (<40%)	−0.02	−0.02
Green tea	−0.02	0.19
Oolong tea	0.01	0.19
Black tea	−0.03	−0.01
Other tea	0.07	0.15
% Explained Variance (Correlations)		
Food intakes (total)	6.26	6.93
Responses (total)	4.23	1.44
Total cholesterol	10.56 (0.29)	0.47 (0.07)
LDL cholesterol	8.47 (0.27)	1.42 (0.14)
HDL cholesterol	4.04 (0.19)	1.60 (−0.12)
Triglycerides	0.62 (−0.07)	0.14 (0.03)
BMI	0.63 (−0.08)	2.76 (0.17)
Waist circumference	1.07 (−0.10)	2.25 (0.15)

LDL cholesterol: low-density lipoprotein cholesterol; HDL cholesterol: high-density lipoprotein cholesterol; BMI: body mass index; *p* < 0.001 for all Pearson correlations with dietary patterns and responses.

**Table 2:** Baseline characteristics of participants according to the quintiles of the dietary pattern scores in the China Kadoorie Biobank (*n* = 479,207).

	Dietary Pattern-1						Dietary Pattern-2					
	Q1 (Low)	Q2	Q3	Q4	Q5 (High)	*p* for Trend	Q1 (Low)	Q2	Q3	Q4	Q5 (High)	*p* for Trend
*n*	98,107	93,329	95,975	97,110	94,686	-	97,087	97,473	95,512	94,747	94,388	-
Dietary pattern score	−0.87	−0.16	0.16	0.30	0.57	<0.001	−0.36	−0.20	−0.04	0.13	0.47	<0.001
Age, year	50.0	53.1	53.1	52.1	49.7	0.006	58.5	53.5	52.6	49.5	43.6	<0.001
Female, %	13.4	38.2	67.2	79.7	79.9	<0.001	75.4	74.8	61.3	46.5	26.7	<0.001
Urban area, %	8.5	62.6	27.2	29.8	89.3	<0.001	2.8	10.9	38.5	66.4	99.0	<0.001
Southern area, %	0.7	18.0	82.7	98.4	99.6	<0.001	94.8	63.5	41.0	39.9	61.1	<0.001
High school and above, %	40.3	45.4	51.3	50.7	59.4	<0.001	36.7	43.3	49.9	55.9	63.5	<0.001
Household income ≥ 20,000 CNY/year, %	27.0	36.8	41.8	43.6	56.1	<0.001	25.0	32.3	41.3	49.5	57.4	<0.001
Married, %	87.3	89.4	90.9	91.9	92.8	<0.001	85.3	89.8	90.7	92.3	94.1	<0.001
Current smoker, %	34.4	29.1	24.7	22.1	19.7	<0.001	29.7	25.8	26.7	25.7	26.2	<0.001
Weekly drinker, %	35.5	25.9	13.2	7.3	6.6	<0.001	27.8	13.6	12.4	10.4	13.9	<0.001
Physical activity, MET-h/day	22.6	21.1	21.5	21.7	20.5	<0.001	23.2	22.3	21.0	21.1	19.7	<0.001
Energy intake, kcal/day	1441.5	1519.4	1509.1	1475.6	1561.8	<0.001	1429.8	1465.8	1482.9	1520.9	1608.4	<0.001
Family history of diabetes, %	5.4	5.9	6.4	6.2	7.1	<0.001	4.6	5.1	5.9	6.6	7.3	<0.001
BMI, kg/m^2^	23.7	23.8	23.5	23.4	23.4	<0.001	22.9	23.4	23.6	23.8	24.2	<0.001
Waist circumference, cm	80.6	80.6	79.5	79.5	79.4	<0.001	78.1	79.5	79.9	80.4	81.8	<0.001

CNY: Chinese Yuan; MET: metabolic equivalent; BMI: body mass index; Q: quintile; SBP: systolic blood pressure; DBP: diastolic blood pressure. *p* for trend is based on a logistic regression analysis for the categorical variables and a linear regression analysis for the continuous variables, assigning median values to the quintile categories of each dietary pattern.

**Table 3:** Hazard ratios (HRs) and 95% confidence intervals (CIs) of diabetes incidence according to the quintiles of the dietary pattern scores of participants in the China Kadoorie Biobank (*n* = 479,207) ^1^.

	Quintiles of Dietary Pattern Scores					*p* for Trend
	Q1 (Low)	Q2	Q3	Q4	Q5 (High)	
Dietary Pattern-1						
Cases	1635	3361	4820	5300	3561	
Incidence rate (/1000 person/year)	1.54	3.38	4.71	5.06	3.54	
Model 1	1.00 (Reference)	1.17 (1.07, 1.28)	1.24 (1.11, 1.37)	1.24 (1.12, 1.38)	1.15 (1.03, 1.30)	0.024
Model 2	1.00 (Reference)	1.16 (1.06, 1.27)	1.19 (1.07, 1.32)	1.18 (1.06, 1.32)	1.08 (0.96, 1.22)	0.363
Dietary Pattern-2						
Cases	4674	4312	3282	3159	3250	
Incidence rate (/1000 person/year)	4.56	4.10	3.20	3.10	3.21	
Model 1	1.00 (Reference)	1.12 (1.07, 1.17)	1.16 (1.09, 1.23)	1.22 (1.14, 1.32)	1.50 (1.36, 1.64)	<0.001
Model 2	1.00 (Reference)	1.09 (1.04, 1.15)	1.12 (1.05, 1.19)	1.18 (1.09, 1.27)	1.44 (1.31, 1.59)	<0.001

^1^ Quintile 1 is the reference category based on Cox proportional regression models and stratified by year of birth (5 years in each category) and sites (10 region sites). Model 1: adjusted for gender, education attainment (no formal school, primary school, middle school, high school, or college/university), marital status (yes or no), and household income (<2500, 2500–4999, 5000–9999, 10,000–19,999, 20,000–34,999, or ≥35,000 CNY/year). Model 2: Model 1 + smoking status (never, occasional, former, or current), alcohol consumption (never regular drinkers, ex-regular drinkers, occasional drinkers, monthly drinkers, reduced drinkers, or weekly drinkers), physical activity (MET-h/day), total energy intake (kcal/day), and family history of diabetes (yes or no). Q: quintile; CNY: Chinese Yuan; MET: metabolic equivalent.

## Data Availability

The dataset for this study is available at www.ckbiobank.org along with the access policy and procedures.

## Supplementary Materials

The following supporting information can be downloaded at: https://www.mdpi.com/article/10.3390/nu14050980/s1. Table S1: Characteristics of participants in the second resurvey of China Kadoorie Biobank; Table S2: Mean intakes of food and beverage groups according to quintiles of dietary pattern scores in the second resurvey of China Kadoorie Biobank (*n* = 17,318); Table S3: Mean intake of food and beverage groups according to quintiles of dietary pattern scores in the China Kadoorie Biobank (*n* = 479,207); Table S4: Hazard ratios (HRs) and 95% confidence intervals (CIs) of diabetes according to quintiles of dietary pattern scores of participants without diabetes incidence occurring in the first two years of follow-up in the China Kadoorie Biobank (*n* = 473,340); Table S5: Hazard ratios (HRs) and 95% confidence intervals (CIs) of diabetes incidence according to quintiles of dietary pattern-1 stratified by gender, age, region, smoking status, physical activity, BMI, and central obesity groups of participants in the China Kadoorie Biobank (*n* = 479,207); Table S6: Hazard ratios (HRs) and 95% confidence intervals (CIs) of diabetes incidence according to quintiles of dietary pattern-2 stratified by gender, age, region, smoking status, physical activity, BMI, and central obesity groups of participants in the China Kadoorie Biobank (*n* = 479,207).
