# Supplementary material for: The Prospective Associations of Lipid Metabolism-Related Dietary Patterns with the Risk of Diabetes in Chinese Adults
Source: Nutrients. 2022 Feb 25;14(5):980. doi: 10.3390/nu14050980 (PMC8912567; doi:10.3390/nu14050980)
Supplement: Supplementary file 1 [file nutrients-14-00980-s001.zip › nutrients-1590418-supplementary.pdf]

Table S1. Characteristics of participants in the second re-survey of China Kadoorie Biobank (*n* = 17,318)

	**Female (*n* = 10,918)**	**Male (*n* = 6,400)**	**Overall (*n* = 17,318)**
Age, year	58.7	59.9	59.1
Urban area (%)	38.8	40.3	39.3
Southern area (%)	67.4	67.9	67.6
High school and above (%)	40.6	57.0	46.7
Household income ≥ 20,000 CNY (%)	77.7	81.8	79.2
Married (%)	84.5	91.8	87.2
Current smoker (%)	1.7	51.5	20.1
Weekly drinker (%)	2.0	28.9	11.9
Physical activity (MET-h/day)	18.1	19.4	18.6
Energy intake (kcal/day)	1525.1	1862.7	1649.8
Family history of diabetes (%)	9.0	9.8	9.5
BMI (kg/m^2)	24.2	24.0	24.1
Waist circumference (cm)	83.0	86.1	84.1

Abbreviations: CNY, unit of Chinese money Yuan; MET, metabolic equivalent; BMI, body mass index.

Table S2. Mean intakes of food and beverage groups according to quintiles of dietary pattern scores in the second res-urvey of China Kadoorie Biobank (n = 17,318).

**Food and beverage intake, g/week**	**Dietary pattern 1**					**Dietary pattern 2**				
	**Q1 (Low)**	**Q2**	**Q3**	**Q4**	**Q5**	**Q1 (Low)**	**Q2**	**Q3**	**Q4**	**Q5**
Rice	665.3	1410.5	2019.3	1857.9	1618.3	2228.4	1728.2	1361.8	1078.6	1174.6
Wheat	1672.5	720.5	298.7	160.1	148.0	227.5	414.5	656.6	818.2	882.9
Other staples	519.4	357.3	154.9	123.8	209.9	31.2	78.8	218.5	445.0	591.8
Meat	222.7	334.2	347.1	427.2	614.1	331.3	377.7	360.7	379.3	496.4
Poultry	54.3	60.6	79.1	90.2	192.8	35.7	55.8	79.1	100.3	206.0
Fish	64.6	99.1	119.6	135.8	287.3	35.7	74.0	110.0	138.3	348.4
Eggs	253.6	232.1	179.5	161.3	191.7	210.1	190.5	208.8	205.5	203.3
Fresh vegetables	1691.7	1417.4	1490.7	1565.1	1893.0	1120.8	1411.2	1603.3	1728.2	2194.1
Fresh fruit	538.2	506.1	515.6	528.6	701.2	234.2	408.2	529.5	642.6	975.1
Preserved vegetables	103.1	84.5	107.0	93.0	88.1	78.2	91.5	89.2	94.5	122.3
Soybean	173.0	169.4	155.2	161.2	264.6	109.2	152.2	175.0	201.4	285.5
Dairy products	237.2	198.4	199.2	212.5	718.8	163.0	227.3	341.1	367.2	467.5
Beer	12.0	6.3	4.3	2.2	2.7	1.4	1.6	3.0	5.7	15.8
Rice wine	1.4	2.6	3.6	4.5	6.9	2.5	2.1	4.3	3.5	6.5
Wine	0.3	0.3	0.4	0.1	0.5	0.5	0.2	0.2	0.3	0.6
Heavy spirit (≥ 40%)	45.1	29.1	19.3	9.7	8.3	49.9	14.8	13.5	14.6	18.7
Light spirit (< 40%)	9.6	8.4	9.5	4.3	3.6	13.2	6.7	3.9	4.0	7.6
Green tea	7.4	8.4	7.5	6.0	6.7	2.4	4.4	6.2	7.8	15.1
Oolong tea	0.6	0.9	0.5	0.5	1.0	0.0	0.0	0.2	0.5	2.7
Black tea	1.2	1.7	1.1	0.5	0.3	1.2	1.1	0.9	0.7	0.9
Other tea	0.3	0.6	0.4	0.6	2.4	0.1	0.2	0.4	0.7	2.9

Table S3. Mean intake of food and beverage groups according to quintiles of dietary pattern scores in the China Kadoorie Biobank (*n* = 479,207).

**Food and beverage intake, g/week**	**Dietary pattern 1**					**Dietary pattern 2**				
	**Q1 (Low)**	**Q2**	**Q3**	**Q4**	**Q5**	**Q1 (Low)**	**Q2**	**Q3**	**Q4**	**Q5**
Rice	246.8	1029.3	1872.4	2105.5	1656.6	1875.8	1509.6	1183.5	1180.1	1135.7
Wheat	1747.2	1034.7	364.9	134.5	247.9	227.8	842.8	909.9	839.8	728.8
Other staples	524.4	330.2	57.3	35.4	84.9	32.8	62.9	398.2	413.7	134.3
Meat	121.0	293.1	311.6	297.0	472.6	294.5	226.3	213.3	291.8	466.7
Poultry	7.6	69.5	69.4	94.5	200.9	38.6	63.3	57.6	96.5	185.7
Fish	15.0	152.1	129.4	141.8	248.5	76.7	79.3	89.4	143.5	297.4
Eggs	177.3	223.2	172.5	151.0	145.5	165.2	159.0	175.8	167.8	201.3
Fresh vegetables	1549.0	1819.4	1554.4	1516.4	1737.1	1400.2	1421.5	1661.6	1747.2	1948.8
Fresh fruit	310.6	550.5	341.5	265.7	570.2	184.3	317.5	303.8	429.8	803.6
Preserved vegetables	150.3	151.7	164.9	163.2	111.9	132.2	195.4	148.2	137.3	128.5
Soybean	69.2	204.3	168.8	169.2	200.7	129.4	145.8	127.9	166.1	241.1
Dairy products	40.9	316.6	178.0	52.3	353.3	29.5	55.0	136.1	274.2	444.6
Beer	4.5	11.7	2.0	0.9	1.2	0.0	0.1	0.8	3.1	16.4
Rice wine	0.0	1.0	2.8	4.2	8.9	0.3	2.2	4.4	5.7	4.3
Wine	0.0	0.2	0.1	0.1	0.2	0.1	0.0	0.1	0.1	0.1
Heavy spirit (≥ 40%)	28.2	56.7	26.5	5.9	2.6	74.1	13.2	12.0	8.6	10.3
Light spirit (< 40%)	10.3	17.9	10.8	4.7	4.6	14.3	8.1	10.2	9.1	6.3
Green tea	5.2	8.3	8.5	5.8	4.7	5.6	4.4	4.9	5.6	12.1
Oolong tea	0.1	0.2	0.3	0.5	0.9	0.0	0.0	0.0	0.1	1.8
Black tea	0.5	2.4	1.5	0.5	0.3	0.9	2.0	1.5	0.3	0.4
Other tea	0.0	0.0	0.0	0.0	0.1	0.0	0.0	0.0	0.0	0.1

Table S4. Hazard ratios (HRs) and 95% confidence intervals (CIs) of diabetes according to quintiles of dietary pattern scores of participants without diabetes incidence occurring in the first two years of follow-up in the China Kadoorie Biobank (n = 473,340) [1.]

	Quintiles of dietary pattern scores					*P* for trend
	**Q1 (Low)**	**Q2**	**Q3**	Q4	Q5 (High)	
			**Dietary pattern 1**			
Cases	1,548	3,145	4,431	4,734	3,286	
Incidence rate (/1000 person-yr)	1.46	3.17	4.33	4.52	3.27	
Model 1	1.00 (Reference)	1.17 (1.07, 1.28)	1.23 (1.11. 1.37)	1.23 (1.10, 1.37)	1.15 (1.01, 1.29)	0.044
Model 2	1.00 (Reference)	1.15 (1.05, 1.27)	1.19 (1.07, 1.33)	1.17 (1.05, 1.32)	1.07 (0.95, 1.22)	0.432
			**Dietary pattern 2**			
Cases	4,291	3,808	3,036	2,963	3,046	
Incidence rate (/1000 person-yr)	4.19	3.62	2.97	2.91	3.01	
Model 1	1.00 (Reference)	1.13 (1.07, 1.19)	1.19 (1.12, 1.27)	1.27 (1.17, 1.37)	1.54 (1.40, 1.69)	<0.001
Model 2	1.00 (Reference)	1.10 (1.05, 1.16)	1.15 (1.08, 1.23)	1.22 (1.13, 1.32)	1.48 (1.34, 1.63)	<0.001

[1] Quintile 1 is the reference category based on Cox proportional regression models and stratified by year of birth (5 years in each category) and sites (10 region sites). Model 2: adjusted for gender, education attainment (no formal school, primary school, middle school, high school, or college/university), marital status (yes or no), and household income (<2500, 2500-4999, 5000-9999, 10000-19999, 20000-34999, ≥35000 CNY/year), smoking status (never, occasional, former, current), alcohol consumption (never regular drinkers, ex-regular drinkers, occasional drinkers, monthly drinkers, reduced drinkers, weekly drinkers), physical activity (MET-h/day), total energy intake (kcal/day), family history of diabetes (yes or no). Test for trend across quintiles were performed by entering median values of quintile categories of each dietary pattern. Q, quintile; CNY, unit of Chinese money Yuan; MET, metabolic equivalent.

Table S5. Hazard ratios (HRs) and 95% confidence intervals (CIs) of diabetes incidence according to quintiles of dietary pattern-1 stratified by gender, age, region, smoking, physical activity, BMI and central obesity groups of participants in the China Kadoorie Biobank (*n* = 479,207)

	Q1 (Low)		Q2		Q3		Q4		Q5		P_{inter}
	N	HR	N	HR (95% CI)	N	HR (95% CI)	N	HR (95% CI)	N	HR (95% CI)	
**Gender**											
Male	906	1.00	1,714	0.95 (0.83, 1.08)	1,919	1.00 (0.87, 1.16)	1,323	0.99 (0.85, 1.15)	1,364	0.97 (0.82, 1.14)	0.141
Female	729	1.00	1,647	1.24 (1.07, 1.42)	2,901	1.28 (1.08, 1.52)	3,977	1.25 (1.05, 1.49)	2,197	1.12 (0.93, 1.36)	
**Age (years)**											
< 50	714	1.00	1,168	1.08 (0.93, 1.24)	1,345	0.97 (0.82, 1.16)	1,652	1.00 (0.84, 1.20)	1,068	0.93 (0.76, 1.13)	<0.001
≥ 50	921	1.00	2,193	1.24 (1.10, 1.39)	3,475	1.34 (1.17, 1.53)	3,648	1.31 (1.14, 1.50)	2,493	1.18 (1.01, 1.37)	
**Rural**											
No	1335	1.00	1,237	1.33 (1.18, 1.50)	3,767	1.36 (1.18, 1.58)	3,865	1.31 (1.12, 1.52)	481	1.27 (1.06, 1.51)	<0.001
Yes	300	1.00	2,124	1.00 (0.88, 1.13)	1,053	1.01 (0.87, 1.17)	1,435	1.05 (0.89, 1.24)	3,080	0.94 (0.79, 1.11)	
**Southern**											
No	1592	1.00	2,480	1.19 (1.08, 1.31)	578	1.08 (0.93, 1.24)	56	0.94 (0.70, 1.26)	15	1.42 (0.85, 2.38)	0.056

Yes	43	1.00	881	0.96 (0.71, 1.30)	4,242	1.03 (0.76, 1.39)	5,244	1.01 (0.74, 1.36)	3,546	0.93 (0.68, 1.26)	
**Current Smoking**											
No	1,050	1.00	2,225	1.23 (1.10, 1.38)	3,489	1.26 (1.10, 1.44)	4,420	1.23 (1.07, 1.42)	2,864	1.10 (0.94, 1.28)	0.382
Yes	585	1.00	1,136	0.95 (0.81, 1.11)	1,331	1.00 (0.84, 1.19)	880	0.99 (0.82, 1.19)	697	1.02 (0.83, 1.25)	
**Physical activity**											
Low	541	1.00	1,575	1.18 (1.04, 1.35)	1,657	1.20 (1.02, 1.42)	1,527	1.16 (0.98, 1.38)	1,652	1.01 (0.83, 1.23)	
Medium	511	1.00	1,041	1.10 (0.94, 1.30)	1,705	1.12 (0.92, 1.35)	1,773	1.10 (0.91, 1.34)	1,163	1.03 (0.83, 1.28)	0.003
High	583	1.00	745	1.07 (0.88, 1.31)	1,458	1.14 (0.92, 1.42)	2,000	1.19 (0.95, 1.48)	746	1.10 (0.87, 1.40)	
**BMI (kg/m²)**											
< 24	517	1.00	821	1.14 (0.94, 1.38)	1,744	1.21 (0.98, 1.49)	1,982	1.23 (0.99, 1.53)	1,091	1.12 (0.88, 1.42)	
24-28	681	1.00	1,480	1.22 (1.07, 1.41)	2,028	1.24 (1.05, 1.45)	2,293	1.28 (1.09, 1.51)	1,693	1.25 (1.04, 1.51)	0.571
≥ 28	437	1.00	1,060	1.17 (1.00, 1.37)	1,048	1.30 (1.07, 1.57)	1,025	1.24 (1.01, 1.52)	777	1.15 (0.91, 1.45)	
**Central obesity**											
No	325	1.0	795	1.27 (1.02,	1,08	1.35 (1.06,	1,05	1.25 (0.97,	671	1.15 (0.87,	0.350

		0		1.59)	4	1.72)	6	1.60)		1.53)
Yes	1,31<br>0	1.0<br>0	2,56<br>6	1.20 (1.09,<br>1.33)	3,73<br>6	1.26 (1.12,<br>1.42)	4,24<br>4	1.27 (1.12,<br>1.43)	2,89<br>0	1.19 (1.04,<br>1.36)

[1] Quintile 1 is the reference category and the model is adjusted for gender, education attainment, marital status, household income, smoking status, alcohol consumption, physical activity, total energy intake, and family history of diabetes. Q, quintile.

Table S6. Hazard ratios (HRs) and 95% confidence intervals (CIs) of diabetes incidence according to quintiles of dietary pattern-2 stratified by gender, age, region, smoking, physical activity, BMI and central obesity groups of participants in the China Kadoorie Biobank (n=479,207)

	Q1 (Low)		Q2		Q3		Q4		Q5		P inter
	N	HR	N	HR (95% CI)	N	HR (95% CI)	N	HR (95% CI)	N	HR (95% CI)	
**Gender**											
Male	1,500	1.00	1,479	1.15 (1.06, 1.25)	1,248	1.18 (1.07, 1.30)	1,351	1.26 (1.13, 1.41)	1,648	1.40 (1.22, 1.60)	<0.001
Female	3,174	1.00	2,833	1.09 (1.02, 1.16)	2,034	1.07 (0.98, 1.17)	1,808	1.17 (1.05, 1.31)	1,602	1.40 (1.21, 1.61)	
**Age (years)**											
< 50	1,083	1.00	1,553	1.12 (1.02, 1.23)	1,083	1.05 (0.94, 1.18)	1,092	1.14 (1.00, 1.30)	1,136	1.29 (1.10, 1.52)	0.588
≥ 50	3,591	1.00	2,759	1.08 (1.02, 1.14)	2,199	1.15 (1.06, 1.24)	2,067	1.19 (1.08, 1.31)	2,114	1.47 (1.31, 1.66)	
**Rural**											
No	4,538	1.00	3,845	1.09 (1.03, 1.15)	1,582	1.10 (1.02, 1.19)	676	1.22 (1.09, 1.37)	44	1.69 (1.25, 2.28)	0.710
Yes	136	1.00	467	0.96 (0.79, 1.16)	1,700	1.05 (0.88, 1.25)	2,483	1.08 (0.91, 1.30)	3,206	1.29 (1.07, 1.55)	
**Southern**											
No	92	1.00	601	1.07 (0.85, 1.33)	1,228	1.11 (0.90, 1.39)	1,563	1.15 (0.92, 1.43)	1,237	1.39 (1.09, 1.77)	0.236

Yes	4,582	1.00	3,711	1.11 (1.05, 1.17)	2,054	1.15 (1.07, 1.23)	1,596	1.23 (1.12, 1.34)	2,013	1.50 (1.34, 1.68)	
**Current Smoking**											
No	3,474	1.00	3,334	1.09 (1.03, 1.15)	2,500	1.12 (1.03, 1.21)	2,346	1.18 (1.08, 1.30)	2,394	1.47 (1.31, 1.66)	<0.001
Yes	1,200	1.00	978	1.14 (1.04, 1.26)	782	1.12 (1.00, 1.26)	813	1.26 (1.10, 1.45)	856	1.33 (1.13, 1.57)	
**Physical activity**											
Low	1,654	1.00	1,074	1.10 (1.00, 1.20)	1,232	1.07 (0.95, 1.20)	1,404	1.08 (0.95, 1.22)	1,588	1.31 (1.13, 1.53)	
Medium	1,702	1.00	1,371	1.09 (1.00, 1.19)	1,006	1.13 (1.01, 1.26)	1,022	1.30 (1.14, 1.49)	1,092	1.63 (1.38, 1.93)	0.021
High	1,318	1.00	1,867	1.10 (1.01, 1.19)	1,044	1.15 (1.04, 1.29)	733	1.20 (1.05, 1.38)	570	1.38 (1.14, 1.67)	
**BMI (kg/m²)**											
< 24	2,048	1.00	1,575	1.04 (0.96, 1.13)	957	1.10 (0.99, 1.22)	812	1.15 (1.00, 1.32)	763	1.37 (1.15, 1.63)	
24-28	1,852	1.00	1,919	1.05 (0.97, 1.13)	1,481	0.99 (0.90, 1.09)	1,401	0.97 (0.87, 1.09)	1,522	1.16 (1.00, 1.33)	0.002
≥ 28	774	1.00	818	1.00 (0.89, 1.12)	844	0.96 (0.84, 1.10)	946	1.02 (0.87, 1.18)	965	1.02 (0.84, 1.23)	
**Central obesity**											
No	944	1.0	984	1.04 (0.94,	675	1.08 (0.94,	637	1.04 (0.88,	691	1.29 (1.05,	0.122

		0		1.16)		1.24)		1.23)		1.60)
Yes	3,730	1.00	3,328	1.10 (1.04, 1.16)	2,607	1.10 (1.02, 1.18)	2,522	1.17 (1.08, 1.28)	2,559	1.33 (1.20, 1.48)

[1] Quintile 1 is the reference category and the model is adjusted for gender, education attainment, marital status, household income, smoking status, alcohol consumption, physical activity, total energy intake, and family history of diabetes. Q, quintile.

Members of the China Kadoorie Biobank collaborative group

International Steering Committee: Junshi Chen, Zhengming Chen (PI), Robert Clarke, Rory Collins, Yu Guo, Liming Li (PI), Jun Lv, Richard Peto, Robin Walters. **International Co-ordinating Centre, Oxford:** Daniel Avery, Ruth Boxall, Derrick Bennett, Yumei Chang, Yiping Chen, Zhengming Chen, Robert Clarke, Huaidong Du, Simon Gilbert, Alex Hacker, Mike Hill, Michael Holmes, Andri Iona, Christiana Kartsonaki, Rene Kerosi, Ling Kong, Om Kurmi, Garry Lancaster, Sarah Lewington, Kuang Lin, John McDonnell, Iona Millwood, Qunhua Nie, Jayakrishnan Radhakrishnan, Paul Ryder, Sam Sansome, Dan Schmidt, Paul Sherliker, Rajani Sohoni, Becky Stevens, Iain Turnbull, Robin Walters, Jenny Wang, Lin Wang, Neil Wright, Ling Yang, Xiaoming Yang. **National Co-ordinating Centre, Beijing:** Yu Guo, Xiao Han, Can Hou, Jun Lv, Pei Pei, Chao Liu, Canqing Yu. **10 Regional Co-ordinating Centres: Qingdao CDC:** Zengchang Pang, Ruqin Gao, Shanpeng Li, Shaojie Wang, Yongmei Liu, Ranran Du, Yajing Zang, Liang Cheng, Xiaocao Tian, Hua Zhang, Yaoming Zhai, Feng Ning, Xiaohui Sun, Feifei Li. **Licang CDC:** Silu Lv, Junzheng Wang, Wei Hou. **Heilongjiang Provincial CDC:** Mingyuan Zeng, Ge Jiang, Xue Zhou. **Nangang CDC:** Liqiu Yang, Hui He, Bo Yu, Yanjie Li, Qinai Xu,Quan Kang, Ziyan Guo. **Hainan Provincial CDC:** Dan Wang, Ximin Hu, Jinyan Chen, Yan Fu, Zhenwang Fu, Xiaohuan Wang. **Meilan CDC:** Min Weng, Zhendong Guo, Shukuan Wu,Yilei Li, Huimei Li, Zhifang Fu. **Jiangsu Provincial CDC:** Ming Wu, Yonglin Zhou, Jinyi Zhou, Ran Tao, Jie Yang, Jian Su. **Suzhou CDC:** Fang liu, Jun Zhang, Yihe Hu, Yan Lu, , Liangcai Ma, Aiyu Tang, Shuo Zhang, Jianrong Jin, Jingchao Liu. **Guangxi Provincial CDC:** Zhenzhu Tang, Naying Chen, Ying Huang. **Liuzhou CDC:** Mingqiang Li, Jinhuai Meng, Rong Pan, Qilian Jiang, Jian Lan,Yun Liu, Liuping Wei, Liyuan Zhou, Ningyu Chen Ping Wang, Fanwen Meng, Yulu Qin,, Sisi Wang. **Sichuan Provincial CDC:** Xianping Wu, Ningmei Zhang, Xiaofang Chen,Weiwei Zhou. **Pengzhou CDC:** Guojin Luo, Jianguo Li, Xiaofang Chen, Xunfu Zhong, Jiaqiu Liu, Qiang Sun. **Gansu Provincial CDC:** Pengfei Ge, Xiaolan Ren, Caixia Dong. **Maiji CDC:** Hui Zhang, Enke Mao, Xiaoping Wang, Tao Wang, Xi zhang. **Henan Provincial CDC:** Ding Zhang, Gang Zhou, Shixian Feng, Liang Chang, Lei Fan. **Huixian CDC:** Yulian Gao, Tianyou He, Huarong Sun, Pan He, Chen Hu, Xukui Zhang, Huifang Wu, Pan He. **Zhejiang Provincial CDC:** Min Yu, Ruying Hu, Hao Wang. Tongxiang CDC: Yijian Qian, Chunmei Wang, Kaixu Xie, Lingli Chen, Yidan Zhang, Dongxia Pan, Qijun Gu. **Hunan Provincial CDC:** Yuelong Huang, Biyun Chen, Li Yin, Huilin Liu, Zhongxi Fu, Qiaohua Xu. **Liuyang CDC:** Xin Xu, Hao Zhang, Huajun Long, Xianzhi Li, Libo Zhang, Zhe Qiu.
